# Writing Development in DHH Students: A Bimodal Bilingual Approach

**DOI:** 10.1093/deafed/enac045

**Published:** 2022-12-14

**Authors:** Moa Gärdenfors

**Affiliations:** Department of Linguistics, Stockholm University, Sweden

## Abstract

This article describes the lexical and syntactic development of written narratives in 24 deaf and hard-of-hearing (DHH) students aged between 8 and 18 and takes into account how their varying linguistic backgrounds may predict their written performance. All participants use spoken Swedish, but the study also considers their proficiency in Swedish Sign language, which ranged from zero to fluent. Their narrative texts were analyzed in regard to syntax and lexicon, which demonstrated a strong developmental trend in which increased age predicted more complex texts. Age of acquisition did not predict any writing outcome, which is suggested to occur because all participants were exposed to language early, that is, within the critical time window for language acquisition. Sign language proficiency showed a tendency to predict adjective density and number of clauses, which encourages future research in this area, especially since this connection argues for the benefits of early access to a language and the positive relationship between sign language proficiency and writing.

## Introduction

Developing sufficient writing skills is fundamental for successfully expressing thoughts, and for engaging a reader’s interest and attention, and these skills are a necessary competence for academic achievement (Graham & Perin, 2007). A typical characteristic of writing development is that a child writes longer texts with more subordinate clauses. Another developmental sign is that a text becomes lexically richer and denser as the child’s vocabulary increases (Berman & Verhoeven, 2002; Hunt, 1966; Johansson, 2009). Additional developmental trends are associated with the number of adjectives and their positions in terms of whether they use as attributes or predicates (Löhndorf, 2021).

The development of writing proficiency among the deaf and hard-of-hearing (DHH) population that uses hearing technology (such as hearing aids or cochlear implants, CI) is an understudied area, as most previous research has focused on reading comprehension. A recent review of literacy development in DHH population (Mayer & Trezek, 2018) suggested that DHH children have more trouble developing writing skills than reading skills. Literacy is often connected to the development of oral skills, and it is generally accepted that spoken language and phonological awareness are closely related (Dammeyer, 2014 and references therein), which explains why language acquisition and development in the DHH group may proceed differently than in typically hearing children. Reduced auditory input may result in a longer time to comprehend spoken language, and might eventually affect the DHH group’s academic achievements (Arfé et al., 2016; Mauldin, 2019; Yoshinaga-Itano, 2003).

Hall and Dills (2020) argued that researchers should take additional variables into consideration along with the multidimensional linguistic experiences of the DHH group because variables (such as age of acquisition (AoA) or signed languages) may influence literacy outcomes. Many DHH studies have limited themselves to a single estimate of their participants’ modes of communication using a monolingual or inconsequential documentation of language proficiency (Hall & Dills, 2020). Although some studies have reported delays in both reading and writing for the DHH group (see Mayer & Trezek, 2018, for review), other studies that considered the linguistic background of DHH children have demonstrated a positive relationship between bimodal bilingualism (the use of two languages of two modalities, such as a spoken and a signed language) and linguistic outcomes (Hassanzadeh, 2012; Amraei, Amirsalari, & Ajalloueyan, 2017; Goodwin, Davidson, & Lillo-Martin, 2017; Caselli, Pyers, & Lieberman, 2021; Gärdenfors, 2021; Johnson, 2021).

The purpose of this study is, from a bilingual view, to examine how and which factors (such as sign language knowledge, AoA, gender, and age) may influence writing development in DHH children, and to describe the linguistic development of DHH children from a syntactic and lexical perspective, with a particular focus on adjectives. This is done in a cross-sectional study of 24 DHH participants aged between 8 and 18 with different linguistic backgrounds.

To the best of my knowledge, no previous study has described the lexical and syntactic development in writing of sign-spoken bilingual DHH children from such a bilingual view. Following previous findings, this study considered the participants’ linguistic background by including AoA and sign language proficiency as possible predictors of writing outcomes. The justification is that in addition to spoken Swedish, many of the DHH participants in this study have various levels of proficiency in Swedish Sign Language (hereafter referred to as STS for Svenskt Teckenspråk) on a continuum from zero to fluent knowledge.

### Syntactic Development

Increasing text length is one of the most characteristic features of age-related writing development, as a growing vocabulary is mirrored in longer texts. With increasing age, sentences and clauses typically are expanded through longer phrases that include, e.g., adjectives (Johansson, 2009). Text length can be measured in number of words, but to reflect syntactic growth, measures such as phrase length and number of clauses are usually used. An established measure for capturing syntactic development (and to allow for comparisons between speaking and writing) is the T-unit (Hunt, 1966), which has been widely used for decades and has been shown to be a comprehensive tool for measuring grammatical complexity in written texts (Wengelin, 2002). Measuring T-units involves parsing text into the smallest possible independent clauses that consist of one main clause and one or more subordinated clauses or non-clausal structures that are embedded or attached to it. Syntactic complexity is demonstrated by a growing number of words and clauses per T-unit.

The T-unit has been used to measure development in several languages. For example, an extensive review of T-units in written and spoken English (Scott, 1988) showed that T-unit length increases slowly by approximately one word per year between the ages of 9 and 19. A third grade native speaker of English had an average of 7.6 words per T-unit and 5 words per clause, while a 12th grader had an average of 14.4 words per T-unit and 8 words per clause. A cross-sectional study by Johansson (2009) showed a similar developmental trend in spoken and written Swedish among typically hearing students. The number of T-units and clauses were included in measures that were analyzed in 79 students aged 10, 13, 17 and in university students. In written narratives, increase as measured by words in T-units was slower than that found by Scott (1988), from 9.4 words per T-unit and 6.3 words per clause in a third–fourth grader, to 11.5 words per T-unit and 6.7 words per clause in a 12th grader.

A measure similar to the T-unit, called macro-syntagm, has been used in several studies in Sweden (Loman & Jörgensen, 1971). Macro-syntagms were primarily developed to analyze spoken language syntax (including dialogic texts), as this syntax requires a more detailed analysis with stricter criteria than afforded by the T-unit. Schönström (2010) analyzed macro-syntagms in narrative texts by 38 sign-print bilingual (using STS and written Swedish) deaf Swedish children in 5th and 10th grade. Schönström’s data showed a developmental trend concerning the use of clauses between these grades, with participants producing and mastering more and different kinds of subordinated clauses as they got older. However, Schönström reported methodological difficulties when parsing texts into macro-syntagms because the sign-print participants tended to exclude subjects or objects, which is a typical L2 feature among this deaf group.

Breland, Lowenstein, and Nittrouer (2022) reported that a DHH group using cochlear implants (CI) was outperformed by typically hearing peers in terms of morphosyntactic measures, including mean length of utterance (MLU), conjunctions and personal pronouns in their written narratives. Interestingly, the written narratives of the DHH group were less complex than their spoken narratives. The DHH group seemed to have developed a sufficient ability to tell spoken narratives, but in writing, they lacked the necessary “writing voice” that is essential for academic achievement, resulting in a discrepancy between their spoken and written performance. The authors suggested that the DHH individuals could not transfer the complex morphosyntactic patterns from spoken to written narratives. Other studies have reported that DHH groups can compete well with typically hearing controls in spoken narratives, but face a disadvantage in written narratives, in which they produce fewer words and shorter clauses, and have a poorer writing quality (Arfé, 2015; Mayer & Trezek, 2018).

### Lexical Development

When young children start learning to write in primary school (ages 6–9), they are occupied with basic writing activities like recognizing and forming letters, orthography, and spelling. A child’s writing development will be reflected in both the syntax (longer texts, more clauses) and lexicon (more vocabulary rich). These skills will gradually become more and more automated and will often be established during middle school, around the age of 12 (Berninger & Swanson, 1994). Adolescent writers will steadily increase their competence in reading and evaluating their own text on a local (eventually global) level, resulting in more mature and developed texts for the age span 13–16. As children grow, they will increasingly automate low-level processes, including processes like typing/handwriting, spelling (related to morphosyntax) and using punctuation, which, according to theories about working memory, might be a prerequisite for the development for more advanced writing skills (McCutchen, 1994).

Lexical development and texts’ lexical properties have often been described through the measures lexical diversity and lexical density (Johansson, 2009). Lexical diversity measures how varied a writer’s vocabulary is, and typically increases with text length as writers become older (Malvern, Richards, Chipere, & Durán, 2004). Two developmental studies on typically hearing Swedish children and adolescents indicated a trend in which lexical diversity increased throughout school but with a particular escalation between fifth and ninth grades (Johansson, 2009; Löhndorf, 2021). Similar patterns have also been observed for other languages. Berman and Verhoeven (2002) examined the writing development of seven languages among typically hearing children (aged 9–17) (Dutch, English, French, Hebrew, Icelandic, Spanish and Swedish) and noted a sharp lexical diversity and text length increase after the age of 12–13. DHH studies have suggested that lexical diversity is generally lower in these students compared to their typically hearing peers (Asker-Árnason et al., 2012; Singleton et al., 2004).

Lexical density examines how dense a text is by dividing the proportion of content words (nouns, verbs, adjectives, and lexical adverbs) by the total number of words (Halliday, 1985). A text with low lexical density is associated with being more speech-like, and a text with high lexical density is associated with being more like written language (Johansson, 2009). DHH studies have found that DHH individuals generally have a higher lexical density than their typically hearing peers, irrespective of linguistic background (Singleton et al., 2004; Asker-Árnason et al., 2012; Gärdenfors, 2021).

It has been proposed that sign language may affect lexical density (Singleton et al., 2004; Asker-Árnason et al., 2012). Swedish DHH participants using CI (11–19 years) had a significantly higher lexical density in their written narratives than their typically hearing peers, which was interpreted as a result of a less developed grammar (that is, the DHH group used fewer grammatical words) (Asker-Árnason et al., 2012). Participants in this study had a typical language development (with spoken Swedish as their first language), although they were to some degree exposed to STS or sign support. The authors speculated that this exposure could have resulted in a transfer from sign language to spoken Swedish, since sign language consists of a higher proportion of content words (and thus a smaller proportion grammar/function words) (Asker-Árnason et al., 2012).

A second suggestion to why DHH children write with higher lexical density is that higher density occurs because of the hearing loss rather than the presence of sign language. Gärdenfors (2021) conducted a study of Swedish 10–11-year-old bimodal bilingual DHH and children of deaf adults (CODA) students with knowledge of STS and spoken Swedish. The DHH group had significantly higher lexical density than the CODA children, despite the fact that both groups had equivalent linguistic proficiency in STS and spoken Swedish. In addition, a correlation analysis showed a relationship between higher lexical density and hearing loss, but not with sign language knowledge. The results were discussed in the relation to the fact that the DHH children did not have full access to a spoken language, and that this limited speech input led to fewer features typical for spoken language like fillers and grammatical words.

### Adjectives

Increasing use of adjectives in Swedish and English is strongly connected to increasing age of the writer (Hunt, 1966; Löhndorf, 2021). The use of adjectives functions as a lexical sophistication that lengthens clauses (Beers & Nagy, 2011; Ravid & Berman, 2010).

A Swedish large-sized adjective study on typically hearing students was conducted by Löhndorf (2021), who examined adjective and noun development in a collection of 832 texts sampled from Swedish national texts sampled from the end-of-year tests taken by typically hearing Swedish students. The collection consisted of narrative and expository texts written by students between the ages of 9 and 17 years. One finding was that the number of adjectives in written narratives increased steadily with age as children became mature writers. Similar findings were reported for Hebrew-speaking children (Ravid & Levie, 2010) in a study based on 252 written and spoken narratives and expository texts by 63 children, adolescents, and adults. In that study, adjectives were acquired later than nouns and verbs, and a more frequent use of adjectives indicated more complex syntax.

The placement of the adjective relative to the noun can also be linked to developmental patterns (Löhndorf, 2021; Ravid & Berman, 2010). An adjective in Swedish and English can be placed either attributively (“a red car”) or predicatively (“the car is red”). Attributive placement has been shown to be developed later than predicate placement. One explanation is that use of the attributive requires a two-step integration of information that may be cognitively demanding for the youngest children to handle, because they must identify the noun and its meaning, and later restrict/define the noun through an attribute (Ravid & Berman, 2010). However, with training, the ability to use adjectives attributively becomes accessible and automated (Ninio, 2004).

There are few studies of DHH students’ use of adjectives, but those that exist demonstrate that the DHH students write fewer adjectives than their typically hearing peers, and that the vocabulary of this group is less varied (Asker-Árnason et al., 2012; Mayer, Watson, Archbold, Ng, & Mulla, 2016; Vizzi, Angelelli, Iaia, Risser, & Marinelli, 2022).

### Language Acquisition

From the overview presented above, we can identify some variables that may predict successful writing development. Commonly used variables include age and gender, but for the DHH group, AoA, and signing knowledge (as a part of bilingualism) are two additional crucial factors.

One commonly held assumption is that if children do not start to acquire a language soon after birth, there is an increased risk that their literacy and academic achievements will be negatively affected. Research has repeatedly shown a positive relationship between early sign language access and reading and writing outcomes for both sign-print and spoken-sign bilinguals (Hoffmeister, 2000; Hoffmeister, 2018; Kuntze, 2004; Svartholm, 2010; Hassanzadeh, 2012; Dostal & Wolbers, 2014; Amraei et al., 2017; Goodwin et al., 2017; Scott & Hoffmeister, 2017; Caselli et al., 2021; Gärdenfors, 2021; Johnson, 2021). For example, native signers who acquired American Sign Language (ASL) at the age of 0–3 have been shown to outperform their peers who acquired ASL later in life (c.f. Mayberry, 1993; Boudreault & Mayberry, 2006; Cormier, Schembri, Vinson, & Orfanidou, 2012).

The discussion about AoA for sign language is complex: when does the critical period end? Are DHH children unable to communicate until their first contact with a natural language? Koulidobrova and Pichler (2021) have argued that DHH children with late AoA are not completely language deprived during the time between birth and first contact with a language (through contact with signing adults, CI operations, etc.). Instead, this group will develop some linguistic features before acquiring their first natural language, which Koulidobrova and Pichler (2021) named initial systems. These systems include gestures, pointing, home signs or some elements of spoken language. They argue that initial systems should be considered as a child’s L1, and that the first “acknowledged” language should be considered their L2. Still, because initial systems cannot replace the early acquisition of a natural L1, it is important to identify a child’s hearing level as early as possible to provide them with appropriate support to circumvent AoA delay (c.f. Yoshinaga-Itano, Sedey, Coulter, & Mehl, 1998).

Another widely discussed issue is that few DHH children are born into a signing environment, and most will not acquire their first language through auditory input in a similar fashion as their hearing peers, leading to a “late AoA.” One estimate holds that 90–95% of DHH children are born into hearing families (Mitchell & Karchmer, 2004), and many of those families have likely never been in contact with deaf people or a sign language before. There is a risk that a DHH child of typically hearing parents will experience late AoA, which will eventually result in poor academic achievement (Mauldin, 2019; Yoshinaga-Itano, 2003).

Svartholm (2010) highlighted the unique situation in Sweden, which is that few Swedish DHH children suffer from a late AoA. The usual explanation is that there has been a long-standing collaboration (which started in the 1980s) between researchers, teachers, and parents that is based on research on STS, in which hearing parents of DHH children have been offered sign language courses. DHH children in this group have been followed during school, and when they graduated from secondary school, they outperformed (in terms of overall school results) their DHH classmates whose parents had no STS knowledge (Svartholm, 2010).

In the beginning of the 21st century, hearing technologies like cochlear implants, hearing aids, and early hearing screening had breakthroughs, which resulted in DHH individuals gaining the ability to acquire language through auditory input. Today, almost all children born profoundly deaf in Sweden receive cochlear implants between the age of 5 and 12 months (Karolinska University Hospital, 2022), and many of them are able to develop and comprehend spoken language (Socialdepartementet, 2016). As a consequence of CI being so widespread, both DHH children and their parents consider spoken language to be sufficient, and fewer DHH children have learned sign language.

### Bilingual Perspectives

The introduction of cochlear implants does not mean that DHH children can rely exclusively on sound strategies in their language acquisition in writing. The technologies may have auditory limitations, which means that DHH children will have differential opportunities for developing literacy in the same way as peers with typical hearing (Pisoni et al., 2008). Early learning of sign language (that is, not relying exclusively on hearing for language acquisition) has therefore been suggested as a precaution to circumvent the potentially limited input of spoken language (see discussion by Kermit, 2010). This proposal has been reinforced by studies showing that DHH children (with hearing technology) of deaf parents, who received access to sign language before their access to auditory information, outperform their DHH peers with hearing parents in several linguistic arenas, including speech perception, speech production, and language development (Amraei et al., 2017; Davidson, Lillo-Martin, & Pichler, 2014; Goodwin et al., 2017; Hassanzadeh, 2012; Johnson, 2021).

Because the participants of this study are on a continuum from being monolingual (spoken and written Swedish) to being bilingual (spoken and written Swedish together with STS), they have different starting points regarding their linguistic use. A review by Williams and Lowrance-Faulhaber (2018) showed that hearing bilinguals had a similar developmental pattern in writing as did monolinguals. However, even if their development is similar, the process of composition may be different due to the double linguistic and cultural repertoires of the bilinguals. For example, a bilingual speaker of spoken Spanish and English can code-switch as a metalinguistic resource to compose texts. Since bilinguals have tools and resources from two languages, the opportunities for written composition will increase thanks to their bidirectional approach, which is also known as the bilingual advantage. As Williams and Lowrance-Faulhaber (2018) described it, “monolingual children who are learning to write in one language have a smaller range of linguistic resources” (p. 63). More research is needed on the effects of the interaction between sign languages and spoken languages (as a part of bilingualism) in DHH people, as many questions remain unexplored (see Fitzpatrick et al., 2016, for a review).

### The Present Study

While the connection between sign language knowledge and AoA and later academic achievements has been established for DHH children, we know less about how these variables interact with the syntactic and lexical development in written narratives. As syntactic and lexical features have been shown to be indicative of linguistic and writing development among the typically hearing population, this study aims to establish how these factors unfold in the DHH children learning to write.

This study aims to examine, from a bilingual view, how and which factors influence writing development in DHH children, and to describe the linguistic development of DHH children from a syntactic and lexical perspective, with a particular focus on adjectives.

These research questions guide the study:

- How do syntactic features develop with age?
- How do lexical features develop with age?
- Can linguistic background predict syntactic and lexical features of DHH group’s texts? If so, how?

## Method

### Participants

Twenty-four DHH participants (eighteen girls and six boys) between 8 and 18 years old were recruited for this study (Table 1). Table 1 is based on information from a background questionnaire filled out by the participants’ parents and teachers before the data collection started. Sixteen of the participants had their deafness confirmed at birth through Newborn hearing screening (NHS), two had their deafness identified between 1 and 6 months, three at the age of 7–12 months, and three between 12 and 24 months. Unfortunately, the background questionnaire did not address the question of whether the children who had their deafness identified after the hearing screenings actually were born deaf, or had a progressive hearing loss.

**Table 1:** Participant metadata

Age	Gender	HT	Identification of deafness	First implant age	Second implant age	dB without HA/CI	dB with HA/CI	AoA first language	Learned Spoken Swedish	Learned STS	SignRepL2 points (max 4.0 p)	DHH family?	School
8.7	Girl	CI	0 month	9 month	9 month	90–	25–39	1.0 years	1.0 years	1.0 years	2.14	No	Mainstreamed
8.7	Girl	HA	0 month			55–69	40–54	From birth	3.0 years	From birth	3.90	Yes	DHH class
9.2	Girl	HA	0 month			40–54	0–24	From birth	2.0 years	From birth	2.78	Yes	Mainstreamed
9.4	Girl	CI	1–6 month	9 month	2.5 years	90–	25–39	2.0 years	2.0 years	*x*	1.84	No	Mainstreamed
1.7	Girl	CI	13–24 month	2.0 years	3.0 years	90–	25–39	3.0 years	3.0 years	10.7	2.44	No	Mainstreamed
10.7	Boy	HA	0 month			55–69	40–54	1.6 years	From birth	From birth	3.88	Yes	DHH class
11.0	Girl	CI	7–12 month	1.2 years	2.1 years	90–	25–39	From birth	2.0 years	1.6 years	3.54	No	Mainstreamed
11.1	Girl	CI	0 month	1.6 years		90–	25–39	From birth	From birth	From birth	3.86	Yes	Mainstreamed
11.3	Girl	CI	0 month	9 month	9 month	90–	25–39	1.6 years	1.6 years	1.6 years	3.78	No	DHH class
11.4	Girl	CI	0 month	2.2 years	4.5 years	90–	25–39	From birth	3.0 years	From birth	3.80	Yes	Mainstreamed
11.6	Boy	HA	1–6 month			45–55	n/a	From birth	1.0 years	From birth	3.84	Yes	DHH class
12.0	Girl	HA	0 month			70–89	40–54	From birth	1.0 years	From birth	3.98	Yes	DHH class
12.7	Boy	HA	0 month			55–69	40–54	8 month	1.6 years	8 month	3.68	No	DHH class
12.8	Girl	HA	0 month			40–54	25–39	From birth	1.0 years	From birth	3.98	Yes	DHH class
12.8	Girl	HA	0 month			55–69	40–54	From birth	2.6 years	From birth	3.98	Yes	DHH class
12.9	Girl	HA	0 month			70–89	55–69	From birth	1.0 years	From birth	3.92	Yes	Mainstreamed
13.8	Girl	CI	0 month	9 month	2.0 years	90-	25–39	2.0 years	2.0 years	2.0 years	2.80	No	Mainstreamed
14.1	Boy	CI	13–24 month	1.10 years	2.4 years	90–	25–39	1.10 years	2.0 years	1.10 years	2.02	No	Mainstreamed
14.6	Boy	CI	7–12 month	1.3 years	1.11 years	90–	25–39	1.0 years	1.7 years	1 years	2.74	No	Mainstreamed
14.7	Girl	HA	0 month			40–54	25–39	From birth	1.0 years	From birth	3.92	Yes	Mainstreamed
15.0	Girl	HA	0 month			70–89	40–54	From birth	1.0 years	From birth	3.86	Yes	Mainstreamed
17.6	Girl	CI	7–12 month	1.8 years	4.2 years	90–	0–24	2.0 years	2.0 years	*x*	2.24	No	Mainstreamed
18.1	Boy	CI	13–24 month	2.7 years	2.8 years	90–	25–39	3.0 years	3.0 years	4–5 years	3.28	No	DHH class
18.6	Girl	CI	0 month	2.4 years	5.0 years	90–	0–24	1.6 years	2.6 years	1.6 years	3.96	No	DHH class

Eleven participants had moderate to severe hearing loss (40–89 dB). With the two hearing aids that they normally wore, they had mild to moderate hearing levels (25–69 dB). The remaining thirteen participants had their first CI implantation between 9 months and 2.7 years. Twelve of them had two CIs, with the second operation between 9 months and 4.5 years of age. Without CIs, they had profound hearing loss (<90 dB) in both ears, and with CIs, they had typical-hearing to mild hearing loss (0–39 dB) in both ears.

The AoA is defined as the age when the participants learned their first language, regardless if it was spoken Swedish, STS or both simultaneously. If a DHH student was born deaf, had deaf parents, and was exposed for STS from birth, their AoA was set to 0. DHH students with hearing parents usually had a later AoA (e.g., 2 years) presumably because they learned their first language when their parents learned STS, or when they received their CI. Thus, their AoAs were set individually to the appropriate age. The AoA in years and months were converted into decimals in this analysis. In total, fifteen participants had their AoA from birth (0–1.0 year), seven participants had AoA between 1.6 and 2.0 years, and two had AoA at 3.0 years of age.

Twenty-two participants had learned some, or were fluent in, STS, while two had never learned STS. Fifteen participants learned STS between the age of 0 and 1.0, five participants learned STS between 1.6 and 2.0, one learned it between 4 and 5, and one learned it at 10.7 years old. Due to the variation in their sign language knowledge, an STS test called SignRepL2 was set (more information is given about this test below).

Nine participants learned spoken Swedish between 0 and 1.0 years old, nine learned it between 1.6 and 2.0 years old, and six learned it between 2.6 and 3.0 years old. Twelve participants had at least one deaf parent, and twelve had hearing parents. Fourteen participants were attending a mainstreamed class and ten were attending a DHH class. Although their spoken language skills were not tested, according to the background questionnaires answered by their parents and teachers before data collection, all were reported to have mastered age-appropriate spoken Swedish and could both comprehend and produce it on a daily basis.

### Design and Materials

The testing session involved two test instruments, namely a written task performed on a computer, and a Swedish Sign language test. The written task was carried out first. The elicitation material was in the form of a two-page cartoon about the Pink Panther. Participants were asked to retell the cartoon story in writing, using a simple word processor program on a computer. The cartoon story included many colorful details requiring description. These served the purpose of eliciting a varied lexicon, including many descriptive words such as adjectives, which are eligible for this analysis. The material is suitable for both younger and older students, and offered the opportunity to investigate how different lexical and syntactic features may grow with age.

For the Swedish sign language test, the participants were presented with SignRepL2, which is an established STS test developed by Schönström and Holmström (2017) shown to be applicable and useful both for skilled and non-skilled signers. This test has previously been used for educational, developmental, and research purposes, and can detect significant differences between students with different and/or developing signing knowledge, which suggests a high validity (Holmström, 2018; Gärdenfors et al., 2019; Gärdenfors, 2021; Schönström et al., 2021. For this test, participants watched a video in STS consisting of 50 sign language sentences with increasing difficulty. They were then asked to replicate the signs as exactly as possible while being video recorded. The test is able to account for the fact that subjects without any signing knowledge are often able to copy handshapes and movements without understanding the meanings, but these subjects often omit crucial linguistic features such as non-manual signals and adverbial mouthings, which are required for higher points (Schönström & Holmström, 2017). SignRepL2 is measured on a five-point scale (0–4), and the most skilled signers were found to come close to 4.0 points while those without any STS knowledge reached approximately 2.0 points, which shows that the test functioned as expected with this test population.

### Procedure

Prior to data collection, parents or participants over the age of 18 filled out a consent form and questionnaire about language use, hearing background and kind of school (e.g., a school for DHH or mainstreamed school). Data collection took place in quiet rooms at hospitals or schools or, in a few cases, at the participants’ homes. Participants were given instructions in their preferred language (STS, spoken Swedish, or a combination of both) by the author, who was present during the sessions.

Participants were informed that the written texts and STS test were to be done independently during the sessions without support or the possibility to ask questions. They were allowed as much time as they required to complete the writing task.

### Linguistic Analyses

This section covers the methodology of the text analysis, which is presented in Tables 2 and 3. These tables summarize the analytical work, including how the syntactic, lexical contents, and adjectives were counted and measured, as well as which tools were used. For the reader’s convenience, general Swedish examples showing grammatical principles are presented directly as English translations, as the examples are transparent and equivalent between both languages.

**Table 2:** Definitions, examples, and explanations of the syntactic measures and how each measure was analyzed

	Feature	Definition	Examples and/or explanations	Tool
**Syntax**	**Number of words**	Number of words was counted as a measure of the narrative text length. Compounds with incorrect spacing between the constituents (according to Swedish spelling rules) such as *rått fälla* instead of the expected *råttfälla* “mousetrap” were counted as one word.	The lion woke up because he heard something (8 words)	Automatically by CLAN
	**Number of T-units (TU)**	Number of T-units per text. Each text was segmented into T-units as the shortest grammatically allowable sentences: a T-unit is defined as a main clause and its subordinated clause(s) or non-clausal structures that are embedded or attached in it (Hunt, 1966).	TU: The panther went to the door TU: and he opened it. (2 TU)	The T-units were coded manually by the author. CLAN was later used to automatically identify the lengths of the T-units (see below).
	**Number of clauses**	Number of clauses per text (Hunt, 1966).	TU: The panther screamed/because he was in pain (2 clauses in 1 TU)	Automatically by CLAN
	**Words per T-unit**	Number of words per T-unit (Hunt, 1966).	TU: The mouse made a sandwich/and started eating it (9 words per T-unit)	Automatically by CLAN
	**Words per clause**	Number of words per clause.(Hunt, 1966).	TU: The panther slept in the bed/which was his favorite spot (11 words/2 clauses: 5.5 words per clause)	Automatically by CLAN
	**Subordinate clause index**	The number of clauses per T-unit, also referred to as syntactic complexity (Hunt, 1966).	A value of 1.5 means that every second T-unit has a subordinated clause (which corresponds to 50%). A value of 2.7 means that a participant writes a subordinate clause 170% as often as they write a main clause (Hunt, 1966).	Automatically by CLAN

**Table 3:** Definitions, examples, and explanations of the lexical measures and how each measure was analyzed

	Feature	Definition	General examples and explanations	Tool
**Lexicon**	**Lexical diversity**	Lexical diversity is a measure of how varied a writer’s vocabulary is: the higher lexical diversity, the more unique words. The measure used here is called VocD, which is included as an automatic analysis in CLAN. This measure has been developed to mathematically compensate for the variation in text length (which has been a problem for the traditional type–token ratio to measure lexical diversity) (MacWhinney, 2000; Malvern et al., 2004).	Examples from this study: The youngest participant (8.7 years old): 54.9 points A middle aged participant (14.7 years old): 71.4 points The oldest participant (18.6 years old): 118.9 points	Automatically by CLAN
	**Lexical density**	Lexical density is a measure of how dense a text is. A text with higher lexical density is indicated in a text with many content words (lexical words). A text with lower lexical density is indicated in a text with many function words (grammatical words) (Halliday, 1985; Johansson, 2009). The measure is a ratio (percentage) of lexical words (nouns, verbs, adjectives and lexical adverbs) to the total number of words.	Data from this study: 8.7 years old: 51% 14.7 years: 53% 18.6 years: 53%	The functional and content words were manually identified by the author. CLAN then extracted the total number of content words, which were divided by the total number of words to obtain a percentage of content words.
	**Adjective density**	An increasing use of adjectives is an indication of more complex syntax (Löhndorf, 2021; Ravid & Levie, 2010). The measure is expressed as a percentage, obtained by dividing the number of adjectives by the total number of words.	That big house is red.	Manually by the author.
	**Attribute density**	Adjectives or participles placed before a verb, modifying a pro/noun. The measure is expressed as a percentage, obtained by dividing the number of attributes by the total number of words.	A quick car. A burning cowhouse.	Manually by the author
	**Predicate density**	Adjectives or participles placed after a verb. The measure is expressed as a percentage, obtained by dividing the number of predicates by the total number of words.	The house is old. He is irritating.	Manually by the author

To facilitate semi-automatic analyses of the texts, the written texts were manually adapted to the well-established transcription convention CHAT, which enabled the use of the analyses option of the computerized language analysis (computerized language analysis) programs, a well-known tool for analyzing corpora of child language, and they provide excellent tools for qualitative as well as quantitative analysis of, for example, morphology, syntax, and lexicon (MacWhinney, 2000).

## Results

### Writing Outcomes and Statistical Analysis

The participants’ outcomes with means included are shown in Table 4, where the differences in writing at different school stages are shown. The means are age-based on the Swedish school system, which has different stages. Students who are 7–9 years old go to primary school, students between 10 and 12 years go to middle school, students between 13 and 15 years go to secondary school, and students between 16 and 19 go to upper secondary school.

**Table 4:** Possible predictors and writing outcomes

Possible predictors				Syntactic measures						Lexical measures				
Age	Gender	SignRepL2	AoA	Number of words	Number of T-units	Number of clauses	Words per T-unit	Words per clause	Subordinated index	Lexical diversity	Lexical density	Adjective density	Attribute density	Predicate density
8.7	Girl	2.14	0	220	30	46	7.2	4.7	1.5	29.5	0.51	1.4%	0.5%	0.9%
8.7	Girl	3.90	0	294	41	63	7.2	4.7	1.5	54.9	0.46	4.1%	3.1%	1.0%
9.2	Girl	2.78	0	327	48	71	6.8	4.6	1.5	44.6	0.45	1.5%	1.5%	0.0%
9.4	Girl	1.84	2.8	225	31	44	7.3	5.1	1.4	67.6	0.50	2.2%	1.8%	0.4%
**Mean Primary school**				**266.5**	**37.5**	**56.0**	**7.1**	**4.8**	**1.5**	**49.2**	**0.48**	**2.3%**	**1.7%**	**0.6%**
10.7	Girl	2.44	2.0	176	32	50	7.5	4.8	1.6	34.5	0.48	1.1%	0.6%	0.6%
10.7	Boy	3.88	0	243	34	41	5.1	4.2	1.2	38.0	0.53	2.5%	1.6%	0.8%
11.0	Girl	3.54	1.6	269	31	56	9.4	5.2	1.8	45.0	0.46	1.9%	1.5%	0.4%
11.1	Girl	3.86	0	318	46	64	6.9	5.0	1.4	54.1	0.62	5.3%	1.9%	3.5%
11.3	Girl	3.78	1.6	337	45	68	8.3	5.5	1.5	76.1	0.49	4.5%	3.9%	0.6%
11.4	Girl	3.80	0	353	42	77	8.4	4.6	1.8	38.9	0.58	1.7%	0.6%	1.1%
11.6	Boy	3.84	0	300	34	63	9.2	5.0	1.9	56.0	0.50	2.3%	2.0%	0.3%
12.0	Girl	3.98	0	322	41	63	7.9	5.1	1.5	52.5	0.52	0.9%	0.6%	0.3%
12.7	Boy	3.68	0	297	25	58	11.9	5.1	2.3	57.1	0.49	3.4%	2.4%	1.0%
12.8	Girl	3.98	0	360	30	57	12.0	6.3	1.9	58.1	0.53	2.2%	1.7%	0.6%
12.8	Girl	3.98	0	394	66	99	6.0	4.0	1.5	71.0	0.58	4.6%	3.0%	1.5%
12.9	Girl	3.92	0	299	35	51	8.4	5.8	1.5	56.1	0.49	1.7%	1.0%	0.7%
**Mean middle school**				**305.7**	**38.4**	**62.3**	**8.4**	**5.1**	**1.7**	**53.1**	**0.52**	**2.7%**	**1.7%**	**1.0%**
13.8	Girl	2.80	2.0	230	35	51	6.5	4.5	1.5	45.5	0.53	1.7%	0.4%	1.3%
14.1	Boy	2.02	1.10	315	32	54	9.8	5.8	1.7	53.6	0.50	1.6%	0.6%	1.0%
14.6	Boy	2.74	1.0	357	35	56	9.7	6.1	1.6	62.6	0.52	2.2%	2.2%	0.0%
14.7	Girl	3.92	0	547	50	95	1.6	5.6	1.9	71.4	0.53	5.7%	2.7%	2.9%
15.0	Girl	3.86	0	416	36	70	11.4	5.9	1.9	87.9	0.54	5.5%	3.4%	2.2%
**Mean secondary school**				**373.0**	**37.6**	**65.2**	**9.6**	**5.6**	**1.7**	**64.2**	**0.52**	**3.3%**	**1.9%**	**1.5%**
17.6	Girl	2.24	2.0	560	43	85	12.7	6.5	2.0	85.7	0.50	4.8%	3.4%	1.4%
18.1	Boy	3.28	3.0	694	47	97	14.8	7.1	2.1	79.7	0.46	4.9%	3.3%	1.6%
18.6	Girl	3.96	1.6	580	42	87	13.8	6.7	2.1	118.9	0.53	5.5%	4.7%	0.9%
**Mean upper secondary school**				**611.3**	**44.0**	**89.7**	**13.8**	**6.8**	**2.1**	**94.8**	**0.50**	**5.1%**	**3.8%**	**1.3%**

A backward stepwise multiple regression analysis using R (R Core Team, 2022) was set to identify possible predictors based on the participants’ linguistic backgrounds (Tables 5 and 6). The independent variables were SignRepL2, gender, AoA, and age. The dependent variables were number of words, number of T-units, number of clauses, number of words per T-units, number of words per clause and subordinate clause index, lexical diversity, lexical density, adjective density, attribute density, and predicate density. At each step, the variable with the highest p-value was removed until the last model showing the variable(s) with a value of less than *p* < .1, which is not significant, but could show a strong tendency.

**Table 5:** A backward regression analysis of text length using the following possible predictors: SignRepL2, Gender, Age of Acquisition (AoA) and Age was set on Number of words, Number of T-units, Number of clauses, Words per T-unit, Words per clause, and Subordinate Index

	First model		Final model			
Possible predictors	beta	*p*	beta	*p*	*F*(df)	*R* ^2^
**Number of words**					46.74 (1.22)	.665
SignRepL2	.723	.170				
Gender	−16.156	.672				
AoA	3.307	.454				
Age	35.103	<.001^*^^*^^*^^*^	37.633	<.001^*^^*^^*^^*^		
**Number of T-units**	Not statistically significant		Not statistically significant			
SignRepL2	.067	.271				
Gender	−6.54	.149				
AoA	.119	.815				
Age	.714	.375				
**Number of clauses**					8.75 (2.21)	.403
SignRepL2	.181	.060^*^	.152	.052		
Gender	−5.360	.433				
AoA	.564	.473				
Age	3.165	.017^*^^*^	3.410	.002^*^^*^^*^		
**Words per T-unit**					37.44 (1.22)	.613
SignRepL2	.0116	.310				
Gender	1.140	.181				
AoA	.066	.490				
Age	.636	<.001^*^^*^^*^^*^	.732	<.001^*^^*^^*^^*^		
**Words per clause**					32.61 (1.22)	.579
SignRepL2	−.001	.950				
Gender	.167	.557				
AoA	.004	.838				
Age	.217	<.001^*^^*^^*^^*^	.225	<.001^*^^*^^*^^*^		
**Subordinate clause index**					12.99 (1.22)	.343
SignRepL2	.005	.087^*^				
Gender	.185	.099^*^				
AoA	.0117	.353				
Age	.0424	.040^*^^*^	.059	.0015^*^^*^^*^		

^*^ *p* < .1.

^**^ *p* < .05.

^***^ *p* < .01.

^****^ *p* < .001

**Table 6:** A backward regression analysis using the following possible predictors: SignRepL2, Gender, AoA, and Age was set on Lexical diversity, Lexical density, Adjective density, Attribute density and Predicate density

	First model		Final model			
Possible predictors	beta	*p*	beta	*p*	*F*(df)	*R^2^*
**Lexical diversity**					28.3 (1.22)	.543
SignRepL2	.073	.436				
Gender	−9.732	.170				
AoA	0.045	.954				
Age	5.626	<.001^*^^*^^*^^*^	5.411	<.001^*^^*^^*^^*^		
**Lexical density**	Not statistically significant		Not statistically significant			
SignRepL2	.011	.459				
Gender	−.021	.325				
AoA	−.001	.571				
Age	.003	.4.69				
**Adjective density**					6.681 (2.21)	.331
SignRepL2	1.84e–04	.0619	1.482e-04	.063		
Gender	−4.66e-03	.5056				
AoA	6.50e–04	.4203				
Age	2.60e–03	.005^*^^*^	2.935e-03	.008^*^^*^^*^		
**Attribute density**					8.144 (1.22)	.237
SignRepL2	1.40e–04	.0621				
Gender	−2.54e–04	.9616				
AoA	6.80e–04	.2703				
Age	1.64e–03	.0970	0.002	.009^*^^*^^*^		
**Predicate density**	Not statistically significant		Not statistically significant			
SignRepL2	4.45e–05	.451				
Gender	−4.40e–03	.316				
AoA	−2.99e–05	.952				
Age	9.58e–04	.230				

^*^ *p* < .1.

^**^ *p* < .05.

^***^ *p* < .01.

^****^ *p* < .001

### Statistical Models of Syntactic Features

A backward stepwise multiple linear regression was set on Text length, including Number of words, Number of T-units, Number of clauses, Words per t-unit, Words per clause, and Subordinate clause index for the possible predictors SignRepL2, Gender, AoA, and Age (Table 5).

Number of words was predicted by age (*p* < .001^*^^*^^*^), which means that word production increased with age. The overall model was *F*(1.22) = 46.74, *p* < .001^*^^*^^*^ with an explained variance of 66.5%. Number of words is equal to −124.54 + 37.63 (age). Number of clauses was predicted by age (*p* = .002^*^^*^) and sign language (*p* = .052.), which means that the older the writer and the higher the points for SignRepL2, the more clauses they produced. The overall model was *F*(2.21) = 8.756, *p* = .002^*^^*^ with an explained variance of 4.3%. Number of clauses is equal to −3.20 + 3.41 (age) + .152 (SignRepL2). Words per T-unit was predicted by age (*p* < .001^*^^*^^*^), which means that the older the writers were, the more words per T-units they produced. The overall model was *F*(1.22) = 37.44, *p* < .001^*^^*^^*^) with an explained variance of 61.3%. Words per T-unit was equal to −.150 + .733 (age). Words per clause was predicted by age (*p* < .001^*^^*^^*^), which means that the older the writers were, the more words per clauses produced. The overall model was *F*(1.22) = 32.61, *p* < .001^*^^*^^*^) with an explained variance of 57.9%. Words per clauses was equal to 2.473 + .225 (age). Subordinated clause index was predicted by age (*p* < .001^*^^*^^*^), which means that the older the writers were, the higher the subordinated clause index. The overall model was *F*(1.22) = 12.99 *p* = .0015^*^^*^) with an explained variance of 34.3%. The subordinated clause index was equal to .938 + .059 (age).

In sum, almost every examined measure of text length showed a strong development with age. The exception is the number of T-units, which did not show any difference with age. Signing knowledge showed a strong predictive tendency for number of clauses (*p* = .052), which means that the more skilled the signers, the greater number of clauses produced.

### Statistical Models of Lexical Features

A backward stepwise multiple linear regression was set on lexical diversity, lexical density adjective density, attribute density, and predicate density on the possible predictors SignRepL2, gender, AoA, and age (Table 6). Lexical diversity was predicted by age (*p* < .001^*^^*^^*^), which means that the older the writer, the higher the lexical diversity. The overall model was *F*(1.22) = 28.3, *p* < .001^*^^*^^*^ with an explained variance of 54.3%. The lexical diversity is equal to −8.421 + 5.411 (age). Adjective density was predicted by age (*p* = .008^*^^*^) and SignRepL2 (*p* = .063), which means that the older the writer and the higher the signing skills, the more adjectives produced. The overall model was *F*(2.21) = 6.681, *p* = .008^*^^*^ with an explained variance of 33.1%. Adjective use was equal to −.031 + .003 (age) + .0001 (SignRepL2). Attribute density was predicted by age (*p* = .009^*^^*^), which means that the older the writer, the more attributes produced. The overall model was *F*(1.22) = 8.144, *p* = .009^*^^*^ with an explained variance of 23.7%. Attribute use was equal to −.008 + .002 (age), meaning that the older the writer, the more attributes they will use. Neither lexical density nor predicate density was predicted by any variable.

In sum, lexical diversity, adjective density, and attribute density showed a strong development with age. In addition, signing knowledge had an effect on the use of adjectives, indicating a possible relation between more skilled signers and a greater number of adjectives in the texts. No variable predicted predicate density or lexical density.

## Discussion

Beyond the individual differences that are a recurrent phenomenon reported in many DHH studies, the statistical analysis showed that age was a very strong predictor of several features of more advanced writing, while gender and AoA did not predict any writing outcome. There was no statistically significant relationship between sign language skills and writing outcomes, but some strong tendencies were revealed, which will be discussed below.

### How Do Syntactic Features Develop with Age?

Age was the strongest predictor of increased text length: the older DHH participants produced longer texts with more words, longer T-units, and more clauses, and demonstrated syntactic development. Increasing age has previously been associated with improved language skills in written Swedish due to training, writing experience, and maturity related to a general language development (cf. Asker-Árnason et al., 2012; Johansson, 2009; Löhndorf, 2021). This study found that the DHH group follows the same pattern with longer and more complex texts with increasing age, and demonstrating development in literacy and linguistics beyond the initial learning to read and write. Developmental studies on Swedish typically hearing children by Johansson (2009) and Löhndorf (2021) showed that 16–17-year old students wrote texts that were three times longer texts than 10–11-year old students, and the acceleration in text length started after the age of 13. To put this into a comparative context, the 17–18-year old DHH participants in this study wrote texts that were twice as long as that of their 10–12-year old peers. The developmental escalation appeared to happen between middle and secondary school, especially between the ages of 14–15, indicating that DHH individuals have a developmental pattern similar to their typically hearing peers, but delayed for a year. The development continued until upper secondary school.

The T-unit analysis of the DHH group in this study deserves a comment. As previously mentioned, Schönström (2010) attempted a macro-syntagm analysis of deaf sign-print Swedish children, and reported that subject and/or object omissions were recurrent phenomena in the constructions of these children, resulting in some difficulties with segmenting those constructions into macro-syntagms. The current study did not explicitly address the subject/object deletion phenomena, but no equivalent segmentation problems were detected during manual coding of the texts into T-units (that is, these participants did not omit subject and object to the same extent that the deaf sign-print group did in Schönström’s study). This observation may indicate simply that these DHH participants had different behaviors concerning the use of subject and/or object. Future analysis and comparison of the studies’ data and materials might reveal more about these observations.

Unexpectedly, age did not predict the number of T-units, which is surprising given the results from earlier studies in which the number of T-units was a strong developmental factor. The difference between this study and previous ones may be the form of the elicitation material. The cartoon consisted of 31 pictures, and the participants—irrespective of age—produced just over one T-unit per image, which accounts for the similarity in number of T-units. However, with increasing age, the T-units expanded with more words, phrases, and clauses, resulting in longer, more advanced, and complex T-units. In terms of actual numbers, the primary school students (8–9 years old) produced 7.1 words per T-unit and 4.8 words per clause, and the upper secondary school students (17–18 years old) produced 13.5 words per T-unit and 6.8 words per clause. These measures correspond to an approximate .7 word increase per T-unit per year, which is similar to the result of an extensive review of 1,900 American students with English as L1 (9–19 years of age) that reported a one word increase per T-unit per year (Scott, 1988).

The use of subordinated clauses also increased steadily with age, from an average of 1.5 clauses per T-unit (one clause per second T-unit) in the youngest DHH participants to approximately 2.1 clauses per T-unit in the eldest DHH participants (just over one clause per T-unit). The large developmental leap in the DHH children seems to occur around the age of 14, which is about one year later than their Swedish and international typically hearing peers (Berman & Verhoeven, 2002; Johansson, 2009; Löhndorf, 2021).

One reason for this developmental leap may be that the DHH participants have, at this point, automated many of their low-level processes (finding keys on the keyboard, spelling, punctuation, etc.). This automation has freed up much of their cognitive capacity, and they can instead focus on high-level processes (reading, evaluating content, global revisions, etc.), which in turn paves the way for longer and more complex texts (Berninger & Swanson, 1994; McCutchen, 1994). The explanation for why the DHH students have a delayed developmental leap is probably multifold and complex, but one possibility is that they have experienced a certain limitation in language input (due to hearing loss, AoA, etc.) compared to their typically-hearing peers.

To summarize the first research question, the text length and syntactic complexity show a general increase in most measures as DHH children mature. These measures follow the same distinct developmental trend that has been reported among typically-hearing children, including a developmental leap that happens roughly at the age of 14. Experiencing the developmental leap seems to occur roughly a year later in DHH children than their typically-hearing peers.

### How Do Lexical Features Develop with Age?

Parallel to syntactic development, the acquisition of lexicon is crucial to linguistic development. Here, the lexical measures consisted of lexical diversity, lexical density, and adjective use (including exploring the attribute and predicate use). Lexical diversity, adjectives, and attribute adjectives were predicted by age, while lexical density and predicates were not.

Because lexical diversity is a measure of how varied a writer’s vocabulary is, it tends to increase with age. Studies have shown a trend for lexical diversity and use of adjectives to increase throughout school, but with a developmental leap between 5th and 9th grades in both Swedish and non-Swedish typically-hearing children (Berman & Verhoeven, 2002; Johansson, 2009; Löhndorf, 2021). This study found a similar trend of lexical diversity and number of adjectives increasing with age, once again with a marked increase after the age of 14, indicating that the participants’ texts became more varied in terms of lexicon, and include more descriptive elements, with increasing age.

Adjectives were included in the analysis because they are a form of lexical sophistication that lengthens clauses (Beers & Nagy, 2011; Ravid & Berman, 2010). Because adjectives are not grammatical requirements, and can often be excluded without resulting in a grammatically incorrect sentence (at least in Swedish), they still serve the essential function of providing deeper meaning to a text. Removing each adjective in a narrative is likely to result in a dry and boring text, and it is crucial to have the competence to use adjectives as descriptors to make a text more interesting.

To the best of this author’s knowledge, no previous study has described the development of adjective use in DHH children. Some studies have reported incidentally that DHH individuals seem to produce fewer adjectives than their typically-hearing peers (Asker-Árnason et al., 2012; Mayer et al., 2016; Vizzi et al., 2022). Unlike the previously mentioned studies, the present study did not include a typically hearing control group, so no direct comparison in adjective use could be made.

Adjectives in the attribute position have been described as being more cognitively demanding to produce than adjectives in the predicative position, which would explain why attributes are rarer among the youngest participants (Löhndorf, 2021; Ravid & Berman, 2010). This study shows that DHH individuals also follow a developmental trend regarding adjective use. The number of adjectives as attributes increased significantly with age, which is in line with the results of studies of the typically hearing population. An increasing use of attributes may indicate that participants have become more able to handle the cognitive demand of two-step integrated information (the process of identifying the noun, and then restricting/defing the noun) that is required by an attribute.

In contrast, the number of predicate adjectives did not change as much with increasing age, and the change was not statistically significant. A qualitative data analysis showed that some participants had difficulties with correct adjective and adverb congruence, such as *han sov djup,* “he slept deep” instead of *han sov djupt*, “he slept deeply.” The grammatical constructions that included incorrect affixes were not numerous, but were noticeable. Similar findings of morphological difficulties have been described among populations of sign-print (Schönström, 2010), sign-spoken bilingual DHH (Goodwin & Lillo-Martin, 2019), and typically hearing L2 learners of Swedish and English (Eklund-Heinonen, 2009).

The second research question focused on the perspectives of lexical content, lexical variation, and the use of adjectives. The overall results are that most measures increase with age, which indicates that the DHH group has integrated and automatized basic linguistic and grammatical knowledge, which have released cognitive space for using more advanced linguistic features, such as increasing lexical richness including adjectives, and attributes. These findings can be seen as further confirmation that, from a developmental perspective, the DHH group does not intermit or deviate in lexical or adjective use.

### Can Linguistic Background Predict Syntactic and Lexical Features of DHH Group’s Texts? If So, How?

Hall and Dills (2020) argued that the linguistic background of DHH students should be taken into consideration when their literacy development is examined. This suggestion helped to motivate the present study to include linguistic background such as sign language proficiency and AoA, to see whether these were predictors of linguistic and writing development.

Sign language proficiency did not significantly predict any of the writing outcomes, which is in line with previous studies that have shown that signing proficiency along with spoken language does not disturb or have a negative influence on lexical and syntactic writing. Nevertheless, there were nearly-significant tendencies for the most proficient signers to produce more clauses and more adjectives (*p* = .052 and .063, respectively). This result is in agreement with previous research showing that early and full access to sign language facilitates the writing and literacy development for both sign-print bilinguals (Hoffmeister, 2000; Hoffmeister, 2018; Kuntze, 2004; Svartholm, 2010; Dostal & Wolbers, 2014; Wolbers, Bowers, Dostal, & Graham, 2014; Scott & Hoffmeister, 2017) and sign-spoken bilinguals (Hassanzadeh, 2012; Amraei et al., 2017; Goodwin et al., 2017; Caselli et al., 2021; Gärdenfors, 2021; Johnson, 2021).

Sign languages are generally described as rich and descriptive, due to their simultaneous use of multimodal resources and spatiality (Johnston, 2012; Liddell, 2003; Ong & Ranganath, 2005), and the most proficient signers may have developed a capability to express similar linguistic variation in writing, for instance by using more adjectives to achieve the “descriptiveness” often reported in sign languages. If true, this explanation would account for the fact that some participants produced more adjective-dense texts through use of their full linguistic repertoire, which in this case included linguistic resources from STS. To put this into context, in Swedish there are the words *mormor* “mother’s mother” and farmor “father’s mother” while English uses the all-embracing word “grandmother.” Potentially, this difference might make it easier for a Swedish speaker to say “my grandmother on my mother’s side” in English, than the simpler “my grandmother.” By analogy, proficient signers may have developed the habit of being more descriptive in written Swedish because they already have the habit of being extra descriptive in STS, thereby conferring a bilingual advantage (Williams & Lowrance-Faulhaber, 2018).

It is crucial to consider the impact of sign language on DHH individuals because this group’s spoken language acquisition does not proceed in the same way as it does in typically-hearing children. Even if DHH children receive early auditory intervention in the form of hearing screenings, CI operations, hearing technology, and intensive speech training, they still take a longer time to comprehend spoken language because their auditory input is reduced (Yoshinaga-Itano, 2003; Arfé et al., 2016; Mauldin, 2019). This delay will likely influence writing performance, especially if a great part of their linguistic repertoire relies on spoken language only. In this study, the bilingual participants that were fluent in STS may have compensated for the longer time needed to comprehend spoken language by expanding their linguistic repertoire using linguistic resources from STS, which does not rely on hearing ability.

Yet another example of how writing performance is influenced can be illustrated through the DHH group’s generally higher use of content words, which has been observed in previous DHH studies (Singleton et al., 2004; Asker-Árnason et al., 2012; Gärdenfors, 2021; Schönström et al., 2021). The lexical density of the children in this study was not predicted by any single variable, but had the design the study included a control group of typically-hearing children, a difference might have appeared. Previous research has suggested that knowledge of a sign language may increase lexical density because sign languages are more compact and employ fewer grammatical words (Singleton et al., 2004). But as mentioned in the introduction, a feasible suggestion was given by Gärdenfors (2021), who suggested that it might be the hearing loss itself that could result in higher lexical density, as spoken languages generally are less dense compared to written languages. This study included both CODA and DHH children, but the DHH children still demonstrated significantly higher lexical density despite the fact that both groups had similar backgrounds in spoken Swedish and STS. This study’s finding was strengthened by the negative correlation that was shown between lexical density and hearing loss, but there was no correlation between lexical density and signing knowledge at all.

Since function words in spoken Swedish (and in spoken English) are more unstressed than content words, a suggestion is that the DHH group does not always comprehend these words that may be reflected in their writing. To test this idea, the texts were manually scanned, and the impression was that the DHH group used fewer verb particles than their typically-hearing peers, which may be one reason why their lexical density is generally higher. Taken together, the DHH students’ hearing loss may result in limitations in transferring spoken language patterns into writing, which in turn leads to a higher lexical density.

Age of acquisition (AoA), which is the other linguistic variable shown to be fundamental for success in L2 learning and future academic achievement (Cormier et al., 2012; Mayberry, 2007), did not predict any writing outcome. The reason may be that all participants started acquiring their first language very early, between 0 and 3 years old with a mean age of 8 months. This age span has been identified as crucial for developing native-like linguistic knowledge, and individuals with AoA before the age of 3 have been reported to outperform peers with later AoA (Mayberry, 1993; Mayberry & Lock, 2003). Based on previous AoA studies (cf. Boudreault & Mayberry, 2006; Cormier et al., 2012; Mayberry, 2007, 2012), it is plausible that the AoA effect would have been a stronger predictor if some participants had had a later AoA.

The early AoA of the group likely derives from the unique situation in Sweden, which is that there are early hearing screenings, early CI operations, and sign language courses are provided to hearing parents of deaf children (also pointed out by Schönström & Hauser, 2021) as soon as their children’s hearing levels are identified. These three factors were also confirmed by the background questionnaires. Several hearing parents reported that even if many of them primarily used spoken Swedish at home with their DHH children, they used supported signs, gestures, and pointing to communicate with their DHH children before they started being able to communicate with them using spoken language. This finding is in line with those of Koulidobrova and Pichler’s (2021) study about pre-lingual communication, and it is likely that the children were provided some linguistic input earlier than reported in the background questionnaire.

### Limitations of the Study

Three methodological caveats could be mentioned concerning (1) the regression model, (2) lack of certain auditory information, and (3) difficulties in identifying the AoA.

The first caveat is that the sample of participants (24) may be on the lower limit for multiple regression analyses, increasing the risk of type 2 errors. However, the results seem quite robust and, considering the aim of the study and the limited number of the population, I feel confident in using this statistical model. It could also be noted that the outcomes are in line with several findings from previous studies.

The second caveat is that proficiency in spoken Swedish was not tested for all participants, and therefore spoken language proficiency could not be included as a variable in this study. This is a serious limitation, not the least when considering Hall and Dills’ (2020) call to include as much linguistic information as possible about DHH participants. The empirical factor behind this limitation is that the participant collection was carried through collaboration with CI teams and through the deaf community (through schools, networking, etc.). The initial plan had been to collect the majority of the participants through the CI teams who, in addition, would provide auditory and linguistic backgrounds through standardized tests (such as vocabulary, audiograms, and spoken comprehension in both calm and noisy environments). However, the participation rate was much lower through the CI teams (8%) than by appeals made directly to the deaf community (75%). This lack of CI team participation meant that most of the desired auditory information was simply not available other than what could be derived from the background questionnaires.

The third caveat concerns the background questionnaire. Parents were asked to state when their children learned STS and spoken Swedish, and these values were subsequently used as the predictor AoA. In retrospect, the question of the time of acquisition of STS/spoken Swedish could have been formulated differently, encouraging parents to indicate when the child was first exposed to language of any kind. For example, a child may have received its first CI at the age of 9 months, but the parents reported that the child started learning both spoken Swedish and STS at age 2, which was when the child started producing the first words and signs. Based on the parents’ answers to the questionnaire, the AoA for that child would be set to 2 years. It is unlikely that the child did not have any language input at all before this age, but the questionnaire did not account for such a case.

It is also problematic that parents interpreted and answered the questions in different ways, which affects the appropriateness of directly comparing the participants. For instance, some parents reported that their DHH children started signing from birth, resulting in their AoAs being set to 0 years, but this does not mean that these children actually started producing signs as soon as they were born. Koulidobrova and Pichler’s (2021) discussion about initial systems is relevant here. Many of the participants with “late AoA” (close to 3 years) may in fact have had an earlier AoA through home signs, gestures or some auditory input before they started producing spoken Swedish or STS by themselves. Consequently, the AoA of the group is likely to be have been even lower than what was reported in this study, and so AoA was possibly even less of a predictor than reported here.

## Conclusion

This study confirmed that DHH children follow the same developmental trends in writing as typical-hearing children, which is that they wrote more complex and mature texts as they grew older. The texts also became richer in vocabulary, including an increased use of adjectives, and especially adjectives as attributes. The increase in lexicon is a characteristic phenomenon when the syntactic increase occurs, and has been described as a sign of more syntactic complexity (Löhndorf, 2021; Ravid & Berman, 2010). Given the lack of previous research on writing development in Swedish DHH pupils, it was important to establish that there are no signs that this group deviates or intermits in their writing development.

A second finding is that sign language proficiency did not have a negative influence on writing outcomes. On the contrary, there was some positive evidence indicating that the most skilled signers produced more adjectives and a higher number of clauses. This finding agrees with previous studies that have also shown positive correlations between sign language and literacy, and that the greater linguistic repertoire of the DHH group, which includes metalinguistic knowledge from STS, could be leveraged into their writing.

A third result is that AoA to language did not predict any writing outcome. All participants had an AoA between 0 and 3 years (mean 8 months). Their overall early AoA is likely within the critical time window for language acquisition. The early AoA is probably due to Sweden’s generally prompt and systematic interventions for DHH children, such as early hearing screenings, early and free CI operations, sign language courses for parents, and support for bilingual schools.

## Ethical statement

The studies involving human participants were reviewed and approved by the Swedish Ethical Review Authority. Written informed consent for participation in this study was obtained from the participants’ parents or from participants older than 18 years of age.

## Acknowledgement

I want to express my greatest gratitude to my two supervisors, Victoria Johansson and Krister Schönström, for their continuous support, advices, and feedback during this study. I also want to thank the anonymous reviewers for valuable comments and feedback.

## Conflicts of Interest

No conflicts of interest were reported.

## Data Availability

The data underlying this article are available in the article and in its online supplementary material.

## Supplementary Material

Supplementary_data_to_manuscript_enac045
